# Cognitive and academic outcomes in children with chronic kidney disease

**DOI:** 10.1007/s00467-022-05499-0

**Published:** 2022-03-03

**Authors:** Siah Kim, Anita Van Zwieten, Jennifer Lorenzo, Rabia Khalid, Suncica Lah, Kerry Chen, Madeleine Didsbury, Anna Francis, Steven Mctaggart, Amanda Walker, Fiona E. Mackie, Chanel Prestidge, Armando Teixeira-Pinto, Allison Tong, Katrina Blazek, Belinda Barton, Jonathan C. Craig, Germaine Wong

**Affiliations:** 1 Centre for Kidney Research, The Children’s Hospital at Westmead, Sydney, Australia; 2 Sydney School of Public Health, The University of Sydney, Sydney, Australia; 3 Children’s Hospital Education Research Institute, The Children’s Hospital at Westmead, Sydney, Australia; 4 Kids Neuroscience Centre, The Children’s Hospital at Westmead, Sydney, Australia; 5 School of Psychology, The University of Sydney, Sydney, Australia; 6 Child & Adolescent Renal Service, Queensland Children’s Hospital, Brisbane, Australia; 7 Department of Renal Medicine, The Royal Children’s Hospital, Melbourne, Australia; 8 Department of Nephrology, Sydney Children’s Hospital at Randwick, Sydney, Australia; 9 School of Women’s and Child Health, University of New South Wales, Sydney, Australia; 10 Department of Nephrology, Starship Children’s Hospital, Auckland, New Zealand; 11 College of Medicine and Public Health, Flinders University, Adelaide, Australia; 12 Centre for Transplant and Renal Research, Westmead Hospital, Sydney, Australia

**Keywords:** Paediatric, CKD, Cognition, Academic achievement

## Abstract

**Background:**

Few data exist on the cognitive and academic functioning of children with chronic kidney disease (CKD) over the trajectory of their illness. We aimed to determine the association between CKD stages and cognitive and academic performance in children over time.

**Methods:**

We included 53 participants (aged 6–18 years) with CKD stages 1–5 (*n* = 37), on dialysis (*n* = 3), or with functioning kidney transplant (*n* = 22) from three units in Australia from 2015 to 2019. Participants undertook a series of psychometric tests and were invited for repeated assessments annually. We used linear regression and linear mixed models to investigate the effect of CKD stage, adjusted for socioeconomic status.

**Results:**

At baseline, full-scale intelligence quotient (FSIQ) (95%CI) of children on kidney replacement therapy (KRT) was in the low average range (87: 78, 96) and average (101: 95, 108) for children with CKD 1–5. Mean (95%CI) FSIQ, word reading, numerical operations, and spelling scores for children on KRT were 14.3 (− 25.3, − 3.3), 11 (− 18.5, − 3.6), 8.5 (− 17.6, 0.76), and 10 (− 18.6, − 1.3) points lower than children with CKD Stages 1–5. Spelling and numerical operations scores declined by 0.7 (− 1.4, − 0.1) and 1.0 (− 2.0, 0.2) units per year increase in age, regardless of CKD stage.

**Conclusions:**

Children treated with KRT have low average cognitive abilities and lower academic performance for numeracy and literacy compared to both children with CKD 1–5 and to the general population. However, the rate of decline in academic performance over time is similar for children across the full spectrum of CKD.

**Graphical abstract:**

A higher resolution version of the Graphical abstract is available as Supplementary information.

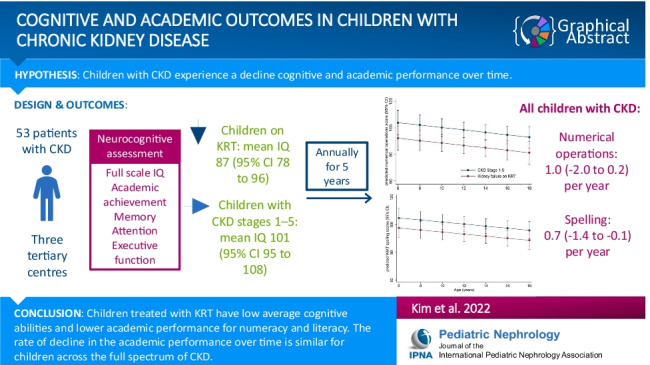

**Supplementary Information:**

The online version contains supplementary material including a graphical abstract available at 10.1007/s00467-022-05499-0.

## Introduction

Chronic kidney disease (CKD) has a major impact on children and their families throughout their life course. Whilst mortality has improved for children with CKD primarily due to successful kidney transplantation [1–5], it is unclear if this has translated into improved long term neurocognitive outcomes for children with CKD. Although there have been recent improvements in the level of adult educational attainment in children with CKD [6], employment levels remain lower compared to peers and also to those with adult onset kidney disease [6, 7].

One of the major contributors to poorer educational and vocational outcomes for children with CKD is cognitive difficulties, with around 50% reporting some difficulty across all stages of CKD [8]. A systematic review of neurocognitive outcomes in children with CKD found global cognition (full scale intelligence quotient; FSIQ) of children with CKD was classified as low average, with mean FSIQ 10.5 points lower than the general population [9]. Mild deficits were observed across the domains of attention, memory, and executive function. Compared with the general population, academic achievement was poorer among children on kidney replacement therapy (KRT), across the domains of reading, mathematics, and spelling [9]. However, most of the included studies evaluating the effects of reduced kidney function on cognition and academic performance were cross-sectional; thus, these studies have provided limited insight into longitudinal changes in cognition and academic skills in children with CKD as their kidney function worsens.

Our study aimed to examine whether cognitive and academic skills of children with CKD stages 1–5 and KRT differ from the general population, and to investigate the effects of CKD stage (CKD stage 1–5 versus KRT) on cognitive and academic outcomes. The study also aimed to test whether there are changes in academic skills of children with CKD over time.

## Methods

A subset of children from the Kids with CKD cohort study (KCAD) were invited to participate in the neurocognitive substudy. As detailed elsewhere, the KCAD study was a cohort study of children with CKD and kidney failure undertaken across five tertiary paediatric hospitals across Australia and New Zealand, with recruitment undertaken from 2012 to 2016 [10, 11]. The neurocognitive study subset included participants from three centres in Australia who were aged 6–18 years, could understand and speak English, and did not have severe visual or hearing impairment that would prevent them from being able to complete study activities. Participants with known intellectual disability were eligible to participate. Participants for the neurocognitive substudy were recruited between 2015 and 2019.

This study was approved by the Human Research Ethics Committee (HREC) of all participating centres (The Children’s Hospital at Westmead and Sydney Children’s Hospital (HREC/12/SCHN/159) and The Royal Children’s Hospital (Royal Children’s Hospital Human Research Ethics Committee: 33,229). Participants and/or their caregivers provided written informed consent (or assent), as appropriate for participant age. This study was conducted in accordance with the Strengthening The Reporting of Observational Studies in Epidemiology Guidelines [12].

### Exposure

The exposure was CKD stage, recorded at the time of each neurocognitive assessment. CKD stage was collected using parent questionnaires and cross checked with the patient’s electronic medical records. Due to the very small number of participants on dialysis, CKD stage was defined as either CKD stages 1–5 or being on KRT including dialysis or kidney transplantation.

### Covariates

At time of baseline assessment for the KCAD study, questionnaires were used to collect the participants’ demographics including age, sex, cause of CKD, time of CKD diagnosis, comorbidities, and ethnicity. Information on learning difficulties was parent reported and obtained through questionnaires. Medical information such as cause of CKD and comorbidities were additionally cross checked with the patient’s electronic medical record. CKD cause was classified on the basis of primary renal disease alone. For socioeconomic status (SES), we collected information from caregivers regarding household income, employment status, educational attainment, perceived financial status, and home ownership and then used principal component analysis to calculate a combined global socioeconomic index score based on these five SES indicators. The global SES index was then divided into quartiles for analysis, with the highest quartile reflecting the highest SES [10].

### Outcomes

Neurocognitive assessment was performed by a qualified psychologist who administered a standardised battery of instruments assessing cognitive and academic skills. Parents of participants who were under 18 years of age completed a standardised questionnaire to assess executive function of the child. Cognitive skills assessed included: intelligence (FSIQ and sub-domains of the Wechsler Intelligence Scale for Children IV: WISC-IV-IV) [13], attention (subtests from the Test of Everyday Attention for Children: TEA-Ch) [14], memory (subtests from the Children’s Memory Scale: CMS) [15] and executive skills (subtests from the Delis Kaplan Executive Function system (DKEFS)) [16], and a parent questionnaire Behaviour Rating Inventory of Executive Function (BRIEF) [17]. Academic skills assessed included: spelling, reading, and numerical operations (subtests from the Wechsler Individual Achievement Test II Australian and New Zealand: WIAT-II II A&NZ) [18]. In all instances, age scaled or age and sex scaled scores were used. Higher scores indicate better skills, aside from the BRIEF where higher scores indicate more difficulties with executive skills in everyday life. Supplemental Table 1 provides a short description of the tests.

Testing was performed in a clinic or hospital environment between 2015 and 2019. The neurocognitive assessment was performed at baseline; then, participants were invited for repeated assessment annually for 4 years. In total, 39 (74%) participants had repeated assessment of academic achievement over time, with 16 (30%), 9 (17%), and 13 (25%) participants having two, three, and four assessments in total respectively. As FSIQ can only be measured biannually due to practice effects, the number of participants with repeated measures of FSIQ was limited (*n* = 18). Therefore, for the longitudinal analysis, we chose to assess academic achievement alone.

### Data analysis

Mean test scores of children with CKD stages 1–5 and those on KRT were compared to population standardised test scores using *z*-tests. Multivariable linear regression was used to investigate the effect of CKD stage on cognitive and academic outcomes. We adjusted for SES in all models given the well-established association between SES and both cognitive and academic outcomes [19–21]. For each of the individual neurocognitive outcomes, we adjusted for age, gender, duration of CKD diagnosis, cause of CKD, and ethnicity if they were found to confound the effect of CKD stage by greater than 10% or were statistically significant with *p* < 0.05. We tested for effect modification between CKD stage and other variables in the final multivariable model, including if they were statistically significant with *p* < 0.01.

To assess whether there were changes in academic achievement over time in children with CKD, we used mixed linear models with a random intercept and coefficient to adjust for the repeated measures for each participant. We tested for effect modification between time and CKD stage and SES. All analyses were conducted using STATA 16.0 (Texas, USA).

## Results

### *Baseline characteristics (Table *1*)*

**Table 1:** Participant characteristics (*n* = 53)

Participant characteristics	Mean (s.d.) or *n* (%)
**Age at baseline assessment (years)**	Mean 12 (s.d. 2.5)
**Length of CKD diagnosis (years)**	Mean 10 (s.d. 3.6)
**CKD stage**	
CKD stage 1–5	27 (52)
Dialysis	3 (6)
Transplant	22 (42)
**CKD Cause**	
Glomerulonephritis	6 (11)
Nephrotic syndrome including FSGS*	16 (30)
CAKUT**	16 (30)
Cystic kidney disease	5 (9)
Other	10 (19)
Socioeconomic status quartile (SES)***	
< 25%	11 (21)
25–50%	11 (21)
50–75%	14 (27)
> 75%	16 (31)
**Ethnicity**	
Aboriginal and or Torres Strait Islander	3 (6)
Middle Eastern	6 (11)
Asian	8 (15)
Anglo/Celtic	31 (58)
Other European	3 (6)
Multi-Ethnic	2 (4)
**Gender**	
Male	37 (70)
Female	16 (30)
**Learning difficulties**	
None	38 (71)
Mild	8 (15)
Moderate	5 (9)
Severe	2 (4)
**Education**	
Local/other school	48 (92)
Home education	1 (2)
Special education local/other school	2 (4)
Special education unit in school	1 (2)
**Language Spoken at Home**	
English	48 (91)
Other	1 (2)
Both English and other	4 (8)

^*^FSGS focal sclerosing glomerulosclerosis **CAKUT congenital anomalies of the kidney and urinary tract. ***SES quartile. Data on CKD cause, CKD stage, SES, learning difficulties, and education were missing for one participant each

**Fig. 1:**
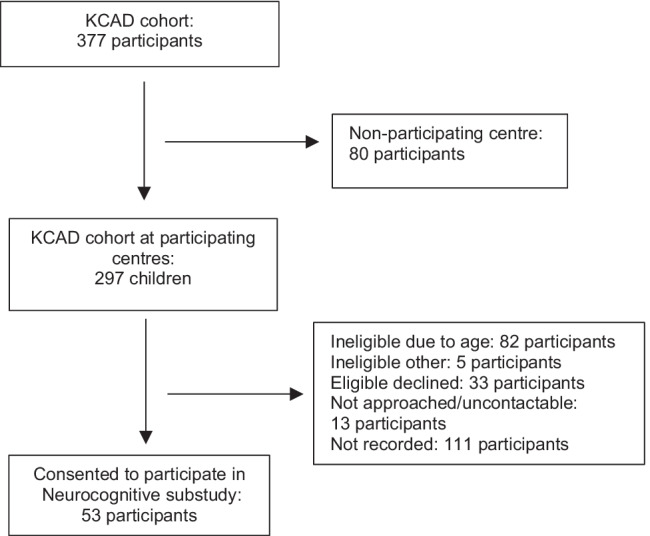
Study recruitment

In total, 53 children participated in the neurocognitive study (Fig. 1). The mean age at first assessment was 12 years (standard deviation 2.5 years), with 37 (70%) male. Twenty-seven (52%) children had CKD stages 1–5, and 25 participants were receiving KRT, with 3 children (6%) treated by dialysis and 22 (42%) with kidney transplants at baseline. The majority of the children had either nephrotic syndrome (*n* = 16, 30%) or congenital anomalies of the kidney and urinary tract (CAKUT) (*n* = 16, 30%) as the cause of their CKD. Thirty-one children (58%) were identified by their caregiver as Anglo/Celtic ethnicity. Children who participated in the study were more likely to be of higher SES with 16 children (31%) within the highest global SES quartile compared to the entire KCAD cohort. Learning difficulties were reported in 15 (28%) of children, with 8 reporting mild difficulties (15%), 5 reporting moderate difficulties (9%) and 2 reporting severe learning difficulties (4%). Within each of the variables of cause of CKD, CKD stage, SES, learning difficulties, and education were missing for one participant each.

### *Baseline cognitive and academic outcomes (Table *2*)*

**Table 2:** Standardised test scores at baseline, stratified by CKD stage

Domain	Standardised tests	Subtest	*n*	CKD Stage 1–5 Mean (95% CI)	Dialysis or transplant Mean (95% CI)
Intelligence	**WISC-IV: Wechsler Intelligence Scale for Children: Fourth Edition**	**Standardised scores: mean 100 s.d. 15**			
		Full scale IQ	49	101 (95, 108)	87 (78, 96)*
		Verbal Comprehension Index	50	99 (91, 106)	94 (87, 101)*
		Perceptual Reasoning Index	50	104 (97, 102)	92 (84, 99)*
		Working Memory Index	50	97 (90, 104)	91 (86, 97)*
		Processing Speed Index	49	100 (94, 106)	93 (86, 100)*
Academic achievement	**WIAT-II: Wechsler Individual Achievement II**	**Standardised scores: mean 100 s.d. 15**			
		Word Reading	49	108 (102, 113)	99 (92, 106)
		Numerical Operations	50	97 (89, 104)	89 (82, 95)*
		Spelling	49	101 (95, 108)	91 (86, 98)*
Attention	**TEA-Ch: Test of Everyday Attention for Children**	**Standardised scores: mean 10 s.d. 3**			
		Auditory sustained attention: Score	48	9.3 (7.8, 10.8)	8 (6.5, 9.4)*
		Visual selective attention: No of correctly identified targets	49	10.8 (9.8, 11.7)	10.4 (9.2, 11.7)
		Visual selective attention: Time per target	49	9.5 (8.6, 10.3)	8.7 (7.3, 10.1)*
		Divided attention: SkySearch	48	8.3 (6.9, 9.7)*	6.3 (4.9, 7.6)*
Memory	**CMS: Children’s Memory Scale**	**Standardised score: mean 10 s.d. 3**			
		Visual/non-verbal memory: Learning	48	10.1 (9.0, 11.2)	10 (8.9, 11.2)
		Visual/non-verbal memory: Total	48	10.6 (9.6, 11.6)	10.5 (9.3, 11.8)
		Visual/non-verbal memory: Long delay	48	11.8 (10.9, 12.7)*	10.8 (9.6, 12.0)
		Auditory/verbal memory: Learning	48	8.6 (7.2, 10.0)*	7.3 (5.8, 8.8)*
		Auditory/verbal memory: Total	48	9.0 (7.4, 10.5)*	7.4 (5.7, 9.0)*
		Auditory/verbal memory: Long delay	48	11.1 (9.7, 12.5)*	8.6 (7.0, 10.3)*
		Auditory/verbal memory: Delayed recognition	48	9.5 (8.1, 10.8)	9.3 (7.8, 10.7)
Executive Function	**DKEFS: Delis Kaplan Executive Function System**	**Standardised score: mean 10 s.d. 3**			
		Verbal fluency: Letter fluency	45	10.9 (9.1, 12.7)	10.1 (8.4, 11.7)
		Verbal fluency: Category fluency	45	11.7 (10.4, 13.0)*	11.0 (9.6, 12.3)
		Verbal fluency: Category switching	45	11.3 (9.7, 13.0)*	12.0 (10.2, 13.7)*
		Verbal fluency: Category switching total switching accuracy	44	11.7 (10.3, 13.1*)	12.2 (10.6, 13.7)*
		Colour word inference: Colour naming	44	10.0 (8.3, 11.8)	9.1 (7.4, 10.7)
		Colour word inference: Word reading	45	11.3 (10.0, 12.6)*	10 (8.7, 11.3)
		Colour word inference: Inhibition	44	10.5 (9.0, 12.1)	9.5 (7.6, 11.4)
		Colour word inference: Inhibition/switching	44	10.3 (8.9, 11.7)	9.5 (7.6, 11.4)
	**BRIEF: Behaviour Rating Inventory of Executive Function**	**Standardised score: mean 50 s.d. 10**			
		Behaviour regulation index	52	53 (48, 57)	56 (50, 62)*
		Metacognition index	52	55 (50, 59)*	59 (54, 63)*
		Global executive composite	52	54 (50, 58)*	58 (53, 63)*

* and bold significant difference to general population using *z*-test (*P* < 0.05). Reasons for incompletion include unable to complete assessment, time constraints, and visual impairment

Compared to the general population test norms, children receiving KRT performed worse across all domains of the WISC-IV, with an average FSIQ of 87 (95% CI 78, 96) in the low average range (defined as FSIQ: 80–89). The average scores across the subdomains were of similar magnitude: verbal comprehension index 94 (95% CI 84, 99), perceptual reasoning index 92 (95%CI 84, 99), working memory index 91 (95% CI 86, 97), and processing speed index 93 (95% CI 86, 100). This was also mirrored with low average scores in the numerical operations and spelling domains of the WIAT-II, with average scores of 89 (95% CI 82, 95) and 91 (95% CI 86, 98). Children with CKD stages 1–5 did not differ across the domains of the WISC-IV or WIAT-II from the general population. Children with parent reported learning difficulties had lower mean scores across FSIQ and academic achievement scores in word reading, numerical operations and spelling (Supplemental Appendix Table S5).

Results from other psychometric testing for attention, memory, and executive skills were more mixed. Using the TEA-Ch, children on KRT had mean scaled scores lower than the general population across tests of both auditory sustained attention (mean 8, 95% CI 6.5, 9.4) and divided attention (mean 6.3, 95% CI 4.9, 7.6). The mean scaled scores of children with CKD stages 1–5 were not significantly different from the general population aside from that for divided attention (mean 8.3, 95% CI 6.9, 9.7). Assessment of memory with the CMS revealed no significant difference in the mean scores on any subtests of visual/ non-verbal memory for children with CKD stages 1–5 or KRT when compared to the general population. However, across the auditory and verbal memory subtests of the CMS, children with CKD stages 1–5 and those treated with KRT performed less well than the general population, with a mean of 9 (95% CI 7.4, 10.5) and 7.4 (95% CI 5.7, 9.0) on the learning and total scores respectively.

On assessment of executive skills, across the conditions of the Colour Word Inference subtest from the DKEFS, scores of children with CKD stages 1–5 and KRT were not significantly different from children within the general population. Children with CKD performed better than the general population within some conditions of the Verbal Fluency subtest. This however was not mirrored by the results of the parent rated BRIEF questionnaire where both children with CKD stages 1–5 and KRT had significantly higher mean scores across the behaviour regulation index, metacognition index, and global executive composite relative to the general population norms, indicating poorer functioning.

### Association between stages of CKD, SES, and cognition and academic achievement at baseline

At baseline, compared to children with CKD stages 1–5, children receiving KRT were found to have a FSIQ 14.3 points lower (95% CI − 25.3, − 3.3) after adjusting for SES, age, and duration since CKD diagnosis (Supplemental Table 2). Across the subdomains of the WISC-IV, there were no significant differences between children on KRT and those with CKD stages 1–5 (Supplemental Table 2). Children on KRT also had WIAT-II word reading scores 11 points lower (95% CI − 18.5, − 3.6), WIAT-II numerical operations scores 9 points lower (95% CI − 17.6, 0.8) and WIAT-II spelling scores 10 points lower (95% CI − 18.6, − 1.3) than children with CKD stages 1–5. Across the cognitive domains of attention, memory, and executive function, scores for children with KRT were not significantly different to children with CKD stages 1–5.

Across most of the domains of cognition and academic achievement, lower SES was associated with poorer cognitive and academic performance compared to children from higher SES backgrounds. This was particularly notable with the WIAT-II, where children in the lowest quartile of SES performed 22 points (95% CI − 33.5, − 11.2), 14 points (95% CI − 27.2, − 0.6), and 15 points (95% CI − 27.1, − 3.0) lower than the highest SES quartile for word reading, numerical operations, and spelling, respectively.

### Changes in academic achievement over time in children across all stages of CKD

Supplemental Table 3 outlines the data structure with respect to repeat psychometric assessments for participants over time. Exactly 39 (74%) of the children had repeat assessments with 16 having two, 9 having three, 13 having four, and one participant with five assessments. Median follow-up for children with repeat assessment was 2.6 years (IQR 1.1–3.7 years).

**Fig. 2:**
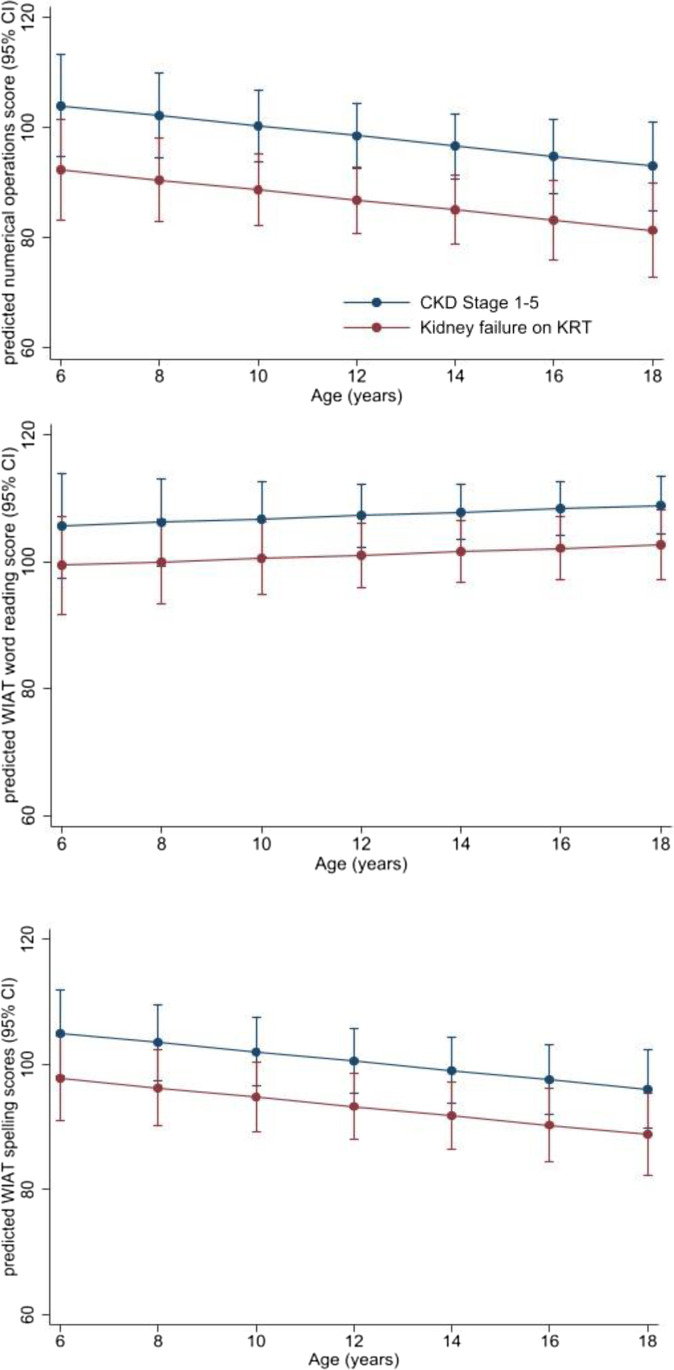
Predicted mean WIAT-II numerical operations, word reading, and spelling scores with increasing age by CKD stage

Results of the multivariable linear mixed model are shown in Table 3 with graphical representation in Fig. 2. Across all stages of CKD, WIAT-II word reading scores did not change with increasing age, with 0.3 point change in scores (95% CI − 0.5, 1.0) for every year increase in age. Children with KRT again had lower word reading scores compared to children with CKD stages 1–5 (− 6.5: 95% CI − 12.3, 0.2), and this difference remained constant with increasing age. There was a trend towards worsening WIAT-II numerical operations scores with age, with a 1 point decline in scores (95% CI − 2.0, 0.2) for every year increase in age. Children with KRT had lower scores in numerical operations compared to children with CKD stages 1–5 (− 11.5: 95% CI − 20.0, − 3.0); however, this difference did not change with age. Children with KRT had lower scores in spelling by 6.4 points (95% CI − 14, 1.2) compared with children with CKD stages 1–5. There was also significant decline in spelling scores with increasing age for all study participants, with a 0.7 point decline for every one year increase in age (95% CI − 1.4, − 0.1). This decline in scores did not differ by CKD stage or SES. We also performed a sensitivity analysis excluding participants on dialysis and found that both the effect of CKD stage and change in scores with age was comparable to the analysis presented above (Supplemental Table 4).

**Table 3:** Longitudinal academic achievement in children with CKD

WIAT-II subtest	Co-variate	Effect on score: β coeff (95% CI)
**Word Reading**	**Age (years)**	0.3 (− 0.5, 1.0)
	**CKD stage**	
	CKD stage 1–5	Ref
	Dialysis and transplant	− 6.5 (− 12.3, 0.2)
	**Socioeconomic status**	
	≥ 75th	ref
	50–75th	− 0.2 (− 8.2, 7.9)
	25–50th	− 14 (− 22.1, − 4.9)
	≤ 25th	− 15.2 (− 25.0, − 5.4)
**Numerical operations**	**Age (years)**	− 0.9 (− 2.0, 0.2)
	**CKD stage**	
	CKD stage 1–5	ref
	Dialysis and transplant	− 11.5 (− 20.0, − 3.0)
	**Socioeconomic status**	
	≥ 75th	ref
	50–75th	9 (− 1.6, 19.6)
	25–50th	− 2.4 (− 14.3, 9.5)
	≤ 25th	− 14.2 (− 26.4, − 2.0)
**Spelling**	**Age (years)**	− 0.7 (− 1.4, − 0.1)
	**CKD stage**	
	CKD stage 1–5	ref
	Dialysis and transplant	− 6.4 (− 13.9, 1.2)
	**Socioeconomic status**	
	≥ 75th	ref
	50–75th	1.7 (− 7.7, 11.1)
	25–50th	− 2.6 (− 14.1, 6.9)
	≤ 25th	− 15.1 (− 25.9, − 4.3)

## Discussion

Using data from a multi-centre longitudinal cohort of children across all stages of CKD, two key findings can be drawn from this study. First, compared to general population norms, children treated with KRT demonstrate worse outcomes in cognition and academic performance. However, a similar association was not observed in children with CKD not yet treated with KRT. At baseline, children treated with KRT had lower overall IQ than the general population, with correspondingly lower scores across all four domains of verbal comprehension index, perceptual reasoning, working memory, and processing speed. This effect was also reflected in reduced academic achievement in numerical operations and spelling. Second, a significant decline in the performance of spelling and numerical operations with increasing age was observed in children across all stages of CKD. Although the yearly decline in scores were small, this may have a cumulative impact on the already poorer academic outcomes for children with CKD.

The magnitude of the effect of KRT on cognition and academic performance is similar to that identified by a previous a systematic review [9]. Most studies reported a 10–15 point reduction in the mean FSIQ for children with KRT compared to children in the general population, placing them in the low average range. However, the influence of kidney transplantation on cognitive and academic outcomes in children with kidney failure remains uncertain. A single study reported kidney transplantation in young children with kidney failure was associated with improvements in cognitive function [22]. Other studies suggest transplantation did not ameliorate poorer cognitive outcomes [22–24]. Our findings suggest children with KRT managed by kidney transplantation continue to have poor cognitive and academic outcomes compared to the general population, even when children on dialysis are excluded. Our limited sample size did not allow us to evaluate differences in cognitive outcomes in children with earlier stage CKD (1–3) and those with advanced stage disease (CKD 4–5) but not yet on dialysis. However, previous studies have reported lower FSIQ score in children with mild-to-moderate CKD compared to the general population [9, 19, 25]. We also found that the effects of CKD on memory and attention were more mixed, with some deficits of auditory memory and visual attention among children receiving KRT. Executive function was worse for children with CKD regardless of stage, compared to the general population, and we found children receiving KRT display more difficulties with executive functions in everyday life; they have poorer behaviour regulation, metacognition and global executive function in contrast to previous reports which found only deficits in metacognition and global executive function [9]. Few studies have assessed the association between baseline CKD stage and later academic outcomes. Our study revealed a decline in both numerical and spelling skills in our cohort of children with CKD of around 1 point per year. Although this was a small change, for a child with score of 100 in Kindergarten, this translates to a score of 90 by year 10 which would place them in the low average range. For children receiving KRT, this decline in numerical and spelling skills has greater clinical significance given the baseline deficits that they already experience and would gradually widen the skills gap with their peers. However, the current findings of the longitudinal change in academic outcomes is largely exploratory. Larger studies over a longer follow up period are needed to explore this trajectory in children with CKD.

Pathways to poor cognitive and academic outcomes in children with CKD are complex and multifactorial. Previous cross-sectional studies highlight absence from school [19] as a major contributor, with a recent study identifying that chronic school absenteeism (defined as missing greater than 18 days of school a year) was present in 17% of children with CKD [26]. Chronic school absenteeism was associated with urological issues, in particular those who needed catheterisation or had enuresis, and larger medication burden [26]. However, severity of kidney disease was not associated with poorer school attendance. Among children with a kidney transplant, one small study identified a mean school attendance of 85%, which was lower than their school peers whose school attendance was 94% [27]. School absteeism has been associated with poorer academic performance regardless of SES [28], and may be a major contributor to poor academic achievement in children with CKD. A qualitative study identified a number of issues regarding school for children with a kidney transplant, namely peer relationship difficulties, difficulty with re-integration, lack of awareness among teachers about their special health needs, and the importance of a hospital–mainstream school liaison [27]. Granular details regarding specifics on schooling, absenteeism and potential educational interventions were not collected in this study. Knowledge of this information will provide important insights into the potential contributors to the poorer academic outcomes in children with CKD. Further studies are needed to evaluate the contribution of healthcare needs, school absenteeism, and education support.

Our study has a number of strengths. The psychometric assessments administered within our study were comprehensive, covering not only IQ but also the specific cognitive domains of language, perceptual-motor function, attention, memory and executive function, and three domains of academic achievement (numerical operations, spelling, reading). We used standardised and validated tests, which were administered by a qualified psychologist. This is one of few studies to repeat assessments of academic performance over time, with 74% of our participants completing repeat assessments. We also had data on relevant clinical and demographic confounders for the models, importantly including a comprehensive assessment of SES.

This study, however, also has a number of limitations. We were unable to investigate the change in cognition over time, with only 18 participants completing follow-up IQ assessments during the study period, and hence the longitudinal effect of CKD on cognition remains unclear and a topic for further research. The sample size of our study is small and therefore has limited power to detect potential differences in cognition and academic achievement between children with and without KRT, and was also unable to determine the contribution of the causes of CKD and duration of CKD on cognition and academic achievement. The median follow-up time was 2.6 years, and may not be sufficient to capture a change across all domains of cognition and academic achievement. However, our study findings are important to inform the sample size calculations and follow-up times of future studies. The high inter-cluster correlation scores (0.8–0.9) indicate that individual participant scores were generally stable over time, suggesting we could potentially lengthen the duration between assessments rather than obtaining more frequent measures of cognition and academic achievement. Paediatric priority access to kidney transplantation commenced in Australia in 2011 with a mean waiting time on dialysis of 1.25 years and consequently our prevalent dialysis population was reduced [29]. For this reason, our recruitment of patients on dialysis was limited and restricted our ability to separately quantify the effect of dialysis on cognition and academic outcomes compared to post-kidney transplantation. Data on the duration of dialysis exposure prior to transplantation was incomplete, and we were unable to specifically investigate how the cumulative effects of uremia may impact cognitive outcomes in children with KRT. Previous studies have shown younger age of transplantation and fewer months on dialysis were associated with better cognitive outcomes [30, 31]. Our study commenced in 2015, and thus, it is likely that many of the children had longer periods on dialysis compared to children currently diagnosed with KRT. The improvement in access to kidney transplantation in children has simultaneously occurred in many countries worldwide, and it would be interesting to see whether this translates into beneficial effects on cognition and academic performance in the future. Nonetheless, the results of our study highlight the need for ongoing surveillance of academic performance in children with KRT despite treatment with kidney transplantation.

In conclusion, our study illustrates that children with KRT have poorer cognitive and academic performance compared to the general population, and academic performance in spelling and numeracy appear to decline over time for all children with CKD. Strategies are needed to improve integration and liaison between education and hospital services, and to improve school attendance for children with CKD.

## Supplementary Information

Below is the link to the electronic supplementary material.

Graphical Abstract 5499 (PPTX 106 KB)

Supplementary file2 (DOCX 44 KB)
