# Supplementary material for: Cognitive and academic outcomes in children with chronic kidney disease
Source: Pediatr Nephrol. 2022 Mar 3;37(11):2715–24. doi: 10.1007/s00467-022-05499-0 (PMC9489550; doi:10.1007/s00467-022-05499-0)
Supplement: Supplementary file 1 — Graphical Abstract 5499 (PPTX 106 KB) [file 467_2022_5499_MOESM1_ESM.pptx]

## Slide 1
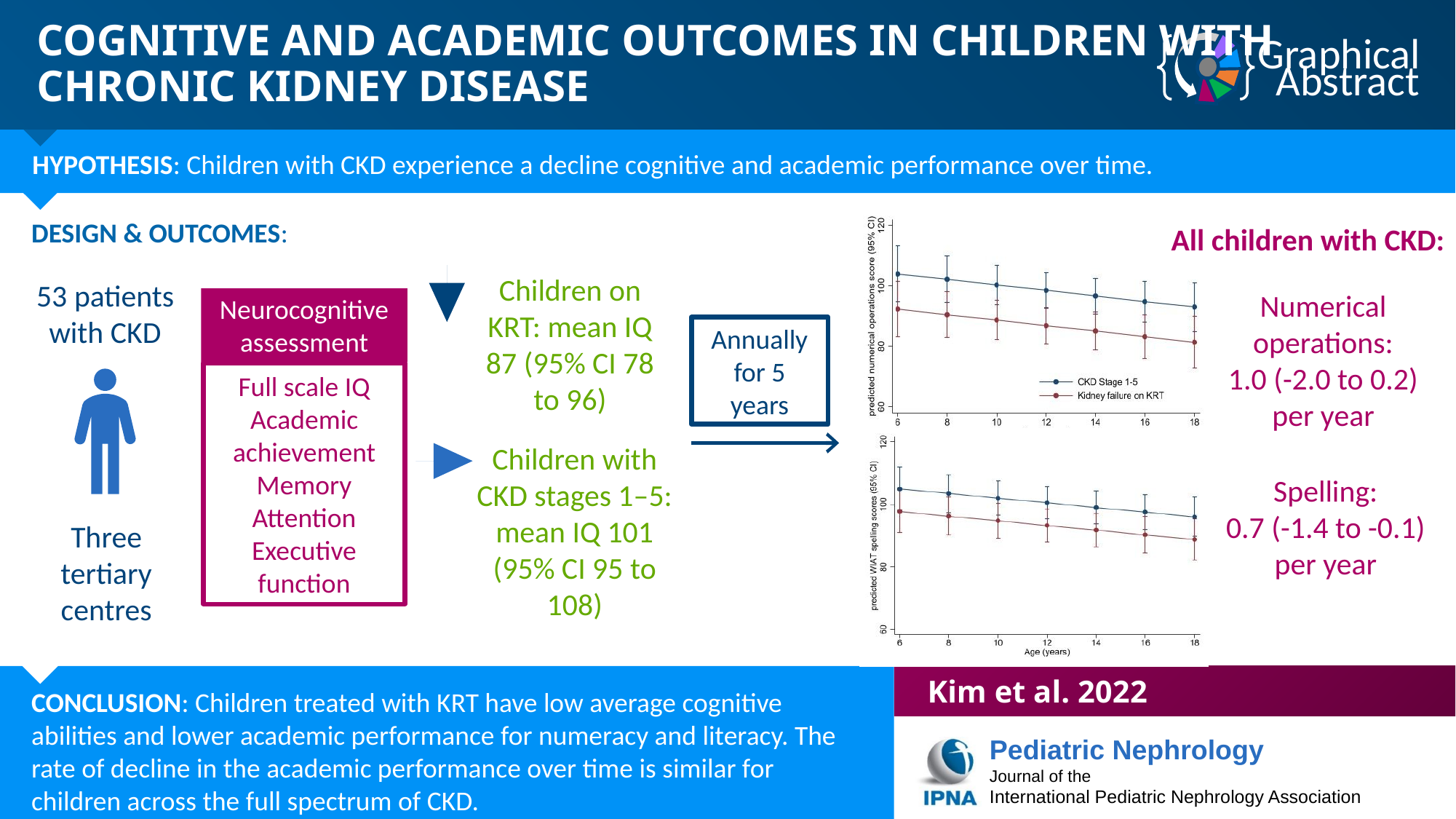

COGNITIVE AND ACADEMIC OUTCOMES IN CHILDREN WITH
CHRONIC KIDNEY DISEASE
HYPOTHESIS: Children with CKD experience a decline cognitive and academic performance over time.
DESIGN & OUTCOMES:
All children with CKD:
Children on KRT: mean IQ 87 (95% CI 78 to 96)
53 patients with CKD
Numerical operations:
1.0 (-2.0 to 0.2) per year
Neurocognitive assessment
Annually for 5 years
Full scale IQ
Academic achievement
Memory
Attention
Executive function
Children with CKD stages 1–5: mean IQ 101 (95% CI 95 to 108)
Spelling:
0.7 (-1.4 to -0.1) per year
Three tertiary centres
Kim et al. 2022
CONCLUSION: Children treated with KRT have low average cognitive abilities and lower academic performance for numeracy and literacy. The rate of decline in the academic performance over time is similar for children across the full spectrum of CKD.
