# Supplementary material for: Cognitive and academic outcomes in children with chronic kidney disease
Source: Pediatr Nephrol. 2022 Mar 3;37(11):2715–24. doi: 10.1007/s00467-022-05499-0 (PMC9489550; doi:10.1007/s00467-022-05499-0)
Supplement: Supplementary file 2 — Supplementary file2 (DOCX 44 KB) [file 467_2022_5499_MOESM2_ESM.docx]

**Supplementary Appendix**

Contents

Supplemental Table 1: Summary and short description of psychometric tests used in the neurocognitive assessment

Supplemental Table 2: Results of multivariable linear regression investigating effect of CKD stage on cognitive and academic domains at baseline assessment

Supplemental Table 3: Data structure of longitudinal assessment

Supplemental Figure 1: Directed acyclic graph for analysis of cognitive and academic outcomes for children with CKD

Supplemental Table 4: Results of sensitivity analysis of longitudinal academic achievement in children with CKD excluding children on dialysis

Supplemental Table 5: Mean FSIQ and academic achievement scores in word reading, numerical operations and spelling for children with learning difficulties

Supplemental Table 1 – Summary and short description of psychometric tests used in the neurocognitive assessment

| Domain | Standardised Tests | Description |
| --- | --- | --- |
| Intelligence | **Wechsler Intelligence Scale for Children: Fourth Edition (WISC-IV IV)**  **Age range: 6–16 years**  **Mean 100 s.d. 15** | The WISC-IV-IV Australian provides composite scores to represent the child’s functioning in four domains:   - Verbal Comprehension Index (VCI), - Perceptual Reasoning Index (PRI), - Working Memory Index (WMI), - Processing Speed Index (PSI).   The WISC-IV-IV Australian also provides a Full Scale Intelligence Quotient (IQ) to represent the child’s overall cognitive ability. |
| Academic achievement | **Wechsler Individual Achievement II (WIAT-II) (selected subsets)**  **Age range: 4–85 years**  **Mean 100 s.d. 15** | Three subtests from the WIAT-II Australian have been selected to assess aspects of academic progress. These are:   - Word Reading: The child is asked to read aloud from a graded word list. - Spelling: The child is asked to write dictated words. - Numerical Operations: The child is asked to solve written calculation problems. |
| Attention | **Test of Everyday Attention for Children (TEA-CH (selected subsets)**  **Age range: 6–15 years**  **Mean 10 s.d. 3** | Three subtests from the TEA-Ch have been selected to assess attention skills.   - Score! – auditory sustained attention - Sky Search – visual selective attention - Sky Search DT – divided attention |
| Memory | **Children’s Memory Scale (CMS) (selected subsets)**  **Age range: 6–16 years**  **Mean 10 s.d. 3** | Two subtests from the CMS have been selected to assess memory.   - Word Pairs (auditory/verbal memory): The child is asked to learn a list of word pairs over three learning trials. - Dot Locations (visual/nonverbal memory): The child is required to learn the spatial location of an array of dots over three learning trials |
| Executive Function | **Delis Kaplan Executive Function System (DKEFS) (selected subsets)**  **Age range: 8–89 years**  **Mean 10 s.d. 3** | Two subtests from the D-KEFS have been selected to assess executive function.   - Verbal Fluency: assesses a child’s ability to generate words fluently in an effortful and phonemic format, from overlearned concepts, and while simultaneously shifting between overlearned concepts. - Color-Word Interference: assesses the inhibition of a more automatic verbal response [reading] in order to generate a conflicting response of naming the dissonant ink colours. |
| Executive Function | **Behaviour Rating Inventory of Executive Function (BRIEF)**  **Age range: 5–18 years**  **Mean 50 s.d. 10**  **Higher scores indicate worse performance** | The BRIEF is a parent questionnaire that assesses executive function behaviour in the home and school environment. The scales combine to form the Behavioral Regulation Index and Metacognition Index, and one composite summary score, the Global Executive Composite. |

Supplemental Table 2: Results of multivariable linear regression investigating effect of CKD stage on cognitive and academic domains at baseline assessment

| Domain | Standardised Tests | Subtest | n | Children with KRT  Beta coeffcient (95%CI) | Other variables in final model * denotes significance | Sensitivity analysis excluding dialysis patients |
| --- | --- | --- | --- | --- | --- | --- |
| Intelligence | **Wechsler Intelligence Scale for Children: Fourth Edition** | Full scale | 47 | **–14.3 (–25.3, –3.3)** | SES* | **–14.3 (–26.5, –2.1)** |
|  |  | Verbal Comprehension | 48 | –4.1 (–13.3, 5.1) | SES* | –3.7 (–13.5, 6.2) |
|  |  | Perceptual reasoning | 48 | –8.6 (–18.5, 1.2) | SES*, age*, CKD time | –7.1 (–17.7, 3.5) |
|  |  | Working memory | 48 | –4.1 (–13.0, 4.9) | SES* | –2.8 (–12.6, 6.9) |
|  |  | Processing speed | 47 | –6.7 (–16.3, 2.8) | SES* | –6.6 (–17.0, 3.7) |
| Academic achievement | **Wechsler Individual Achievement II (WIAT-II II)** | Word Reading | 47 | **–11.1 (–18.5, –3.6)** | SES* CKD cause* | **–9.6 (–17.8, –1.4)** |
|  |  | Numerical operations | 48 | –8.5 (–17.6, 0.76) | SES* | –6.4 (–16.3, 3.4) |
|  |  | Spelling | 47 | **–10 (–18.6, – 1.3)** | SES* | –7.2 (–16.4, 2.1) |
| Attention | **Test of Everyday Attention for Children (TeaCH or TEA)** | Auditory sustained attention | 47 | –1.1 (–3.25, 1.13) | SES | –0.3 (–2.7, 2.0) |
|  |  | Visual selective attention:  No of correctly identified targets | 48 | –0.1 (–1.68, 1.55) | SES | 0.2 (–1.5, 1.9) |
|  |  | Visual selective attention:  Time per target | 48 | –1.0 (–2.5, 0.58) | SES | –0.5(–2.1, 1.1) |
|  |  | Divided attention | 47 | **–2.2 (–4.2, –0.2)** | SES | –2.0 (–4.1, 0.2) |
| Memory | **Children’s Memory Scale (CMS)** | Visual/non-verbal memory: Learning | 47 | 0.3 (–1.2, 1.7) | SES*, CKD cause* |  |
|  |  | Visual/non-verbal memory: Total | 47 | 0.2 (–1.2, 1.7) | SES*, CKD cause* |  |
|  |  | Visual/non-verbal memory: Long delay | 47 | –0.5 (–2.0, 1) | SES |  |
|  |  | Auditory/verbal memory: Learning | 47 | –1.6 (–3.6, 0.5) | SES, ethnicity* |  |
|  |  | Auditory/verbal memory: Total | 47 | –1.8 (–4.1, 0.4) | SES, ethnicity* |  |
|  |  | Auditory/verbal memory: Long delay | 47 | –2.1 (–4.2, 0.1) | SES* |  |
|  |  | Auditory/verbal memory: delayed recall | 47 | –0.4 (–2.2, 1.4) | SES, ethnicity* |  |
| Executive Skills | **Delis Kaplan Executive Function System (DKEFS)** | Verbal fluency: Letter fluency | 43 | 1.5 (–3.8, 0.8) | SES* |  |
|  |  | Verbal fluency: Category fluency | 43 | –1.0 (–2.9, 1.0) | SES |  |
|  |  | Verbal fluency: Category switching total correct | 43 | 1.3 (–1.1, 3.6) | SES, ethnicity* |  |
|  |  | Verbal fluency: Category switching total switching accuracy | 43 | 0.8 (–1.3, 2.9) | SES, ethnicity* |  |
|  |  | Colour word inference: colour naming | 43 | –1.4 (–3.6, 0.8) | SES* |  |
|  |  | Colour word inference: word reading | 43 | –1.0 (–2.7, 0.7) | SES*, CKD time* |  |
|  |  | Colour word inference: inhibition | 43 | –1.1(–3.5, 1.2) | SES* |  |
|  |  | Colour word inference: inhibition/switching | 43 | –0.4 (–2.2, 1.4) | SES*, ethnicity* |  |
|  | **Behaviour Rating Inventory of Executive Function (BRIEF)** | Behaviour regulation index | 50 | 3.2 (–4.2, 10.5) | SES |  |
|  |  | Metacognition index | 50 | 4.6 (–2.1, 11.2) | SES |  |
|  |  | Global executive composite | 50 | 4.1 (–2.6, 10.9) | SES |  |
|  |  |  |  |  |  |  |

* denotes Wald test for covariate P ≤0.05. Bold denotes significant difference from children with CKD stages 1–5.

Supplemental Figure 1: Directed acyclic graph for analysis of cognitive and academic outcomes for children with CKD

CKD Stage

Cognition and academic achievement

Reduced school attendance

Uraemia

Hypertension

Anaemia

Medications (e.g. steroids)

Learning difficulties

Socioeconomic status

Duration of CKD

Ethnicity

Sex

Age

Supplemental Table 3: Data structure of longitudinal assessment

| Frequency | Percent (%) | Pattern of assessments |
| --- | --- | --- |
| 16 | 30 | XX… |
| 14 | 26 | X…. |
| 8 | 15 | XXXX. |
| 5 | 9 | XXX.X |
| 4 | 8 | XX.X. |
| 4 | 8 | XXX.. |
| 1 | 2 | XX..X |
| 1 | 2 | XXXXX |

Supplemental Table 4: Results of sensitivity analysis of longitudinal academic achievement in children with CKD excluding children on dialysis

| WIAT-II subtest | Co-variate | Effect on score: β coeff (95% CI) |
| --- | --- | --- |
| Word Reading | **Age (Years)** | 0.3 (–0.5, 1.0) |
|  | **CKD Stage** |  |
|  | CKD stage 1–5 | ref |
|  | Transplant | –10.7 (–18.3, –3.1) |
|  | **Socioeconomic status** |  |
|  | ≥75^th^ | ref |
|  | 50–75^th^ | 2.3 (–6.9, 11.6) |
|  | 25–50^th^ | –15.3 (–26.1, –4.4) |
|  | ≤ 25^th^ | –17.2 (–27.9, –6.6) |
| Numerical operations | **Age (Years)** | –1.0 (–2.1, 0.1) |
|  | **CKD Stage** |  |
|  | CKD stage 1–5 | ref |
|  | Transplant | –11.3 (–20.2, –2.4) |
|  | **Socioeconomic status** |  |
|  | ≥75^th^ | ref |
|  | 50–75^th^ | 8.9 (–1.9, 19.8) |
|  | 25–50^th^ | –1.9 (–14.8, 10.9) |
|  | ≤ 25^th^ | –14.2 (–26.8, –1.7) |
| Spelling | **Age (Years)** | –0.8 (–1.5, –0.1) |
|  | **CKD Stage** |  |
|  | CKD stage 1–5 | ref |
|  | Transplant | –4.9 (–12.5, 2.6) |
|  | **Socioeconomic status** |  |
|  | ≥75^th^ | ref |
|  | 50–75^th^ | –1.6 (–7.7, 10.8) |
|  | 25–50^th^ | –0.8 (–11.7, 10.0) |
|  | ≤ 25^th^ | –15.2 (–25.7, –4.6) |

Supplemental Table 5: Mean FSIQ and academic achievement scores in word reading, numerical operations and spelling for children with learning difficulties

| Learning difficulties | Full scale IQ  mean (95%CI) | Word Reading  mean (95% CI) | Numerical Operations  mean (95% CI) | Spelling  Mean (95% CI) |
| --- | --- | --- | --- | --- |
| None | 100 (93, 106) | 109 (105, 113) | 97 (91, 102) | 103 (98, 107) |
| Mild | 81 (76, 85) | 87 (76, 97) | 82 (72, 92) | 85 (74, 96) |
| Moderate/Severe | 77 (58, 96) | 90 (84, 97) | 81 (77, 85) | 75 (70, 79) |
